# Preparation of PO_4_^3−^-Intercalated Calcium–Aluminum Hydrotalcites via Coprecipitation Method and Its Flame-Retardant Effect on Bamboo Scrimber

**DOI:** 10.3390/molecules28104093

**Published:** 2023-05-15

**Authors:** Ying Ran, Ailian Hu, Fei Yang, Chungui Du, Jiawei Zhu, Yuran Shao, Yuting Wang, Qichao Bao

**Affiliations:** College of Chemistry and Materials Engineering, Zhejiang A & F University, Hangzhou 311300, China; ry18119295807@163.com (Y.R.); hal15857832323@163.com (A.H.); yangfeier0826@163.com (F.Y.); shao18309819091@163.com (Y.S.); wangyuting270229@icloud.com (Y.W.); bqc1125573308@gmail.com (Q.B.)

**Keywords:** bamboo scrimber, coprecipitation method, calcium–aluminum hydrotalcite, flame retardants, phosphate ion

## Abstract

To improve the flame retardancy of bamboo scrimber, flame-retardant CaAl-PO_4_-LDHs were synthesized via the coprecipitation method using PO_4_^3−^ as the anion of an intercalated calcium–aluminum hydrotalcite in this work. The fine CaAl-PO_4_-LDHs were characterized via X-ray diffraction (XRD), Fourier-transform infrared spectroscopy (FTIR), cold field scanning electron microscopy (SEM), energy-dispersive X-ray (EDX) and thermogravimetry (TG). Different concentrations (1% and 2%) of CaAl-PO_4_-LDHs were used as flame retardants for the bamboo scrimber, and the flame retardancy of the bamboo scrimber was characterized via cone calorimetry. The results showed that CaAl-PO_4_-LDHs with excellent structures were successfully synthesized via the coprecipitation method in 6 h and at 120 °C. Compared with the bamboo scrimber without the flame retardant treatment, the peak heat release rate (HRR) of the bamboo scrimber treated with 1% and 2% concentrations of flame-retardant CaAl-PO_4_-LDHs decreased by 16.62% and 34.46%, the time taken to reach the exothermic peak was delayed by 103 s and 204 s and the Time to Ignition (TTI) was increased by 30% and 40%, respectively. Furthermore, the residual carbon of the bamboo scrimber did not change significantly, increasing by 0.8% and 2.08%, respectively. CO production decreased by 18.87% and 26.42%, respectively, and CO_2_ production decreased by 11.11% and 14.46%, respectively. The combined results show that the CaAl-PO_4_-LDHs synthesized in this work significantly improved the flame retardancy of bamboo scrimber. This work exhibited the great potential of the CaAl-PO_4_-LDHs, which were successfully synthesized via the coprecipitation method and applied as a flame retardant to improve the fire safety of bamboo scrimber.

## 1. Introduction

Bamboo scrimber is a new type of composite bamboo lumber, which is made from bamboo culms crushed into loose reticular fibrous bundles [1,2]. In recent years, bamboo scrimber has been widely used in construction and decoration materials due to its excellent mechanical properties and environmental friendliness [3]. However, bamboo scrimber is a flammable material with significant fire hazards due to the raw material’s nature, which greatly limits its industrial application [4,5]. Therefore, conducting flame retardancy research on bamboo scrimber is of great significance.

In recent years, layered double hydroxides (LDHs) have been emerging as a new generation of environmentally friendly flame-retardant material, which is a new type of layered inorganic functional material made of a positively charged main layer and interlayer region containing charge-compensating anions [6,7]. Its general formula is ${\left[M_{1-x}^{2+}M_{x}^{3+}{\left(OH\right)}_{2}\right]}_{1}^{x+}\;{\left[A^{n-}\right]}_{\frac{x}{n}}\cdot mH_{2}O$, where $M^{2+}$ and $M^{3+}$ denote divalent and trivalent cations located on the main layer, e.g., Mg^2+^, Fe^2+^, Zn^2+^, Al^3+^, Cr^3+^, Fe^3+^, etc. $A^{n-}$ denotes *n* valent interlayer anions, e.g., NO_3_^−^, SiO_3_^2−^, BO_3_^3−^, etc., and *x* denotes the molar ratio of $\frac{M^{3+}}{M^{3+}+M^{2+}}$. The most common $M^{2+}:M^{3+}$ ratios are 2:1 and 3:1 [8,9,10]. LDHs can be prepared via coprecipitation at an adjusted pH followed by the hydrothermal aging of the precipitate [11]. LDHs have been shown to offer excellent flame retardancy and smoke suppression properties due to their unique chemical composition and layered structure. Furthermore, the layer surface of flame-retardant LDHs is abundant in hydroxyl groups, which can form hydrogen bonds with the numerous hydroxyl groups in bamboo, enabling LDHs to stably bind to the bamboo and preventing leaching [12]. Therefore, flame-retardant LDHs can be a useful solution to the current problem of easy leaching in flame retardants.

More importantly, numerous studies have demonstrated that the introduction of new organic anions or functional flame-retardant anions in the interlayer can effectively improve the flame-retardant efficiency of LDHs. For example, Liu et al. [13] investigated the synthesis of sodium dodecylbenzene sulfonate (SDBS)-intercalated aluminum hydroxide via coprecipitation and ion exchange. Xu et al. [14] prepared the triazine-sulphonate pillared layered double hydroxides (LDH-NS). The research results indicate that the thermal stability of LDHs prepared via intercalation is significantly improved. Currently, phosphorus-based flame retardants are non-toxic and low-smoke and are an important part of bamboo flame-retardant research [15,16]. PO_4_^3−^ containing flame retardants will produce PO• free radicals during the combustion process, which can bind H• or HO• in the flame and air, so as to achieve a flame-retardant effect [17]. However, the significant disadvantages of phosphorus-based flame retardants are the complex preparation process, easy leaching and low compatibility. Thus, based on the anion-exchangeable characteristics of LDHs [18], PO_4_^3−^ with flame-retardant effects can be intercalated into the interlayer of LDHs to enhance the flame retardancy.

In addition, the cations on the LDH layer can be replaced by other cations; for example, Damindarova et al. [19] prepared tin–aluminum hydrotalcites and Zhang [20] prepared zinc–aluminum hydrotalcites. There are abundant and inexpensive reserves of calcium and phosphorus in the world, and among available works, one can rarely find a comprehensive report on the flame retardancy of calcium–aluminum hydrotalcites. Thus, Ca^2+^ with flame retardancy can be chosen as a substitute for divalent cations on the LDH layer to prepare flame-retardant calcium–aluminum hydrotalcites with high thermal stability.

In this work, PO_4_^3−^ was selected for the intercalation modification of LDHs containing Ca and Al on the layer. CaAl-PO_4_-LDHs were synthesized via the coprecipitation method under alkaline conditions and applied to bamboo scrimber as flame retardants. The effects of different crystallization temperatures and times on the structure and properties of the synthesized CaAl-PO_4_-LDHs were investigated. Finally, bamboo scrimber was treated with different concentrations of CaAl-PO_4_-LDHs to further investigate the flame-retardant effect of CaAl-PO_4_-LDHs on bamboo scrimber. This work provided a certain theoretical basis for the application of flame-retardant PO_4_^3−^-intercalated calcium–aluminum hydrotalcites to bamboo scrimber.

## 2. Results and Discussion

### 2.1. XRD Analysis of CaAl-PO_4_-LDHs Synthesized via Different Processes

To explore the optimal processes for the preparation of CaAl-PO_4_-LDHs via coprecipitation, the CaAl-PO_4_-LDHs samples were synthesized and characterized at different crystallization temperatures (80 °C, 100 °C and 120 °C) and times (4 h, 6 h and 8 h). The effects of different crystallization temperatures and times on the structure and properties of CaAl-PO_4_-LDHs were investigated.

**Figure 1:**
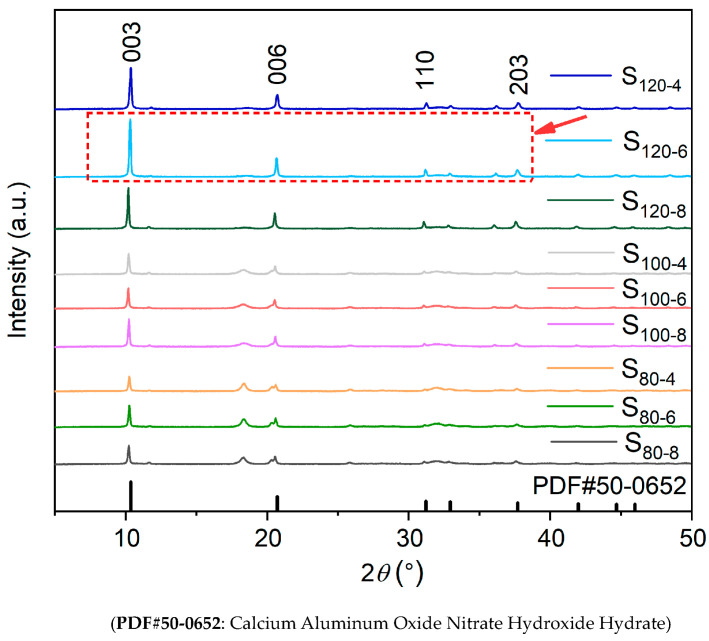
XRD patterns of CaAl-PO4-LDHs prepared at different crystallization temperatures and times.

The XRD patterns of CaAl-PO_4_-LDHs synthesized after crystallization reactions are shown in Figure 1. The main characteristic peaks of CaAl-PO_4_-LDHs display sharp and intense (003), (006), (110) and (203) reflections corresponding to PDF#50-0652, which indicates that the prepared samples had a typical hydrotalcite lamellar structure [21]. The shifts in the diffraction peaks towards lower angles indicate that PO_4_^3−^ was successfully intercalated into the interlayer spacing of calcium–aluminum hydrotalcites [14]. Furthermore, as shown in Table 1, by Bragg’s law (2dsinθ = nλ), the interlayer spacing (d_003_, d_006_ and d_110_) of CaAl-PO_4_-LDHs and PDF#50-0652 were calculated. The characteristic peaks of the samples synthesized at different crystallization temperatures and times were varied. The XRD spectrum shows that the characteristic peak of the sample (S_120-6_) had the sharpest peak shape and the highest intensity. It has been concluded that a synthesis temperature of 120 °C and a duration of 6 h for crystallization are the optimal process parameters for the preparation of CaAl-PO_4_-LDHs.

### 2.2. Effects of Crystallisation Temperature on CaAl-PO_4_-LDHs

According to the XRD results, the effects of crystallization temperature (80 °C, 100 °C and 120 °C) at the optimum crystallization time (6 h) on hydrotalcite samples was investigated. The structure of CaAl-PO_4_-LDHs samples synthesized via the co-precipitation method was characterized via FTIR, SEM, EDX and TG.

**Figure 2:**
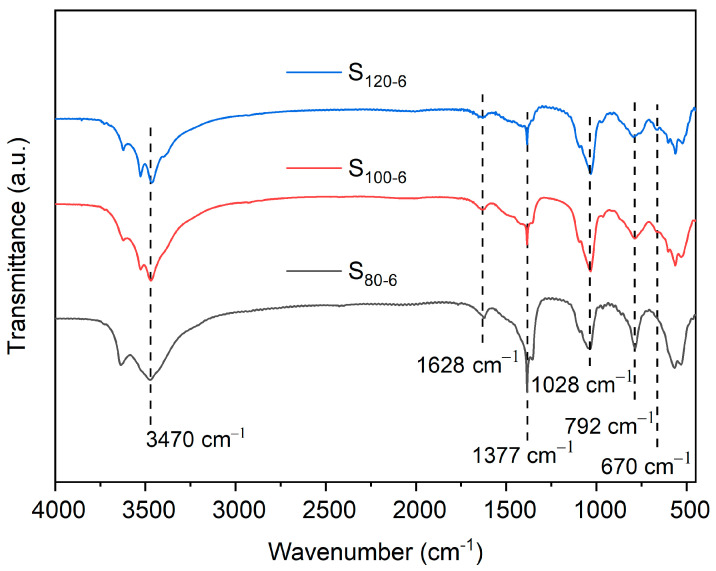
FTIR spectra of CaAl-PO_4_-LDHs prepared at different crystallization temperatures.

Figure 2 shows that the positions of the absorption peaks of FTIR spectra of CaAl-PO_4_-LDHs synthesized at different crystallization temperatures are similar. The signals near 3470 cm^−1^ are related to the stretching vibration of the water molecules in the interlayer [22,23]. The absorption signal at 1628 cm^−1^ corresponds to the H-O-H bending vibration [14]. The characteristic absorption signals of the CO_3_^2−^ group were observed at 1377 cm^−1^ and 792 cm^−1^, which were caused by the adsorption of CO_2_ by CaAl-PO_4_-LDHs after exposure to air at the end of synthesis [24]. The signals below 670 cm^−1^ were attributed to O-M-O bonds and M-O (where M is Ca or Al) bonds [25]. A telescopic vibrational signal of P-OH appeared at 1028 cm^−1^, indicating that PO_4_^3−^ was successfully inserted into the calcium–aluminum hydrotalcites [26].

**Figure 3:**
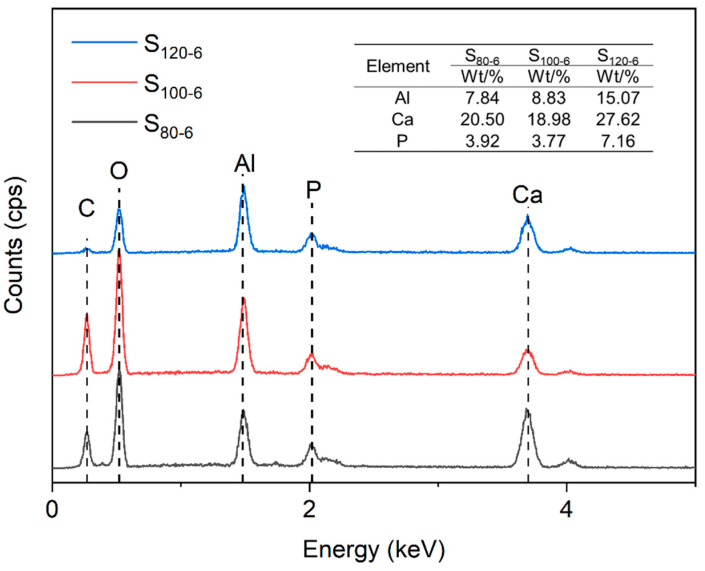
Elemental analysis diagram and element contents of CaAl-PO_4_-LDHs prepared at different crystallization temperatures.

The method of EDX analysis enabled the indirect analysis of the successful insertion of PO_4_^3−^ anions into the interlayer [27], and the results are shown in Figure 3. The ratios of the relative contents of Ca and Al of the CaAl-PO_4_-LDHs samples were close to the theoretical value of 2.0, which indicated the successful synthesis of a typical layered hydrotalcite structure [28]. The content of elemental C in the samples synthesized at 100 °C was significantly higher due to the higher doping of carbonate in the samples. The C element was almost absent in S_120-6_, indicating the oxidation of this sample was less likely to produce CO_3_^2−^. With the increase in the crystallization temperature, the P content first decreased and then increased. The highest elemental P content in the samples was reached at 120 °C. The results indicate the successful synthesis of CaAl-PO_4_-LDHs [29].

**Figure 4:**
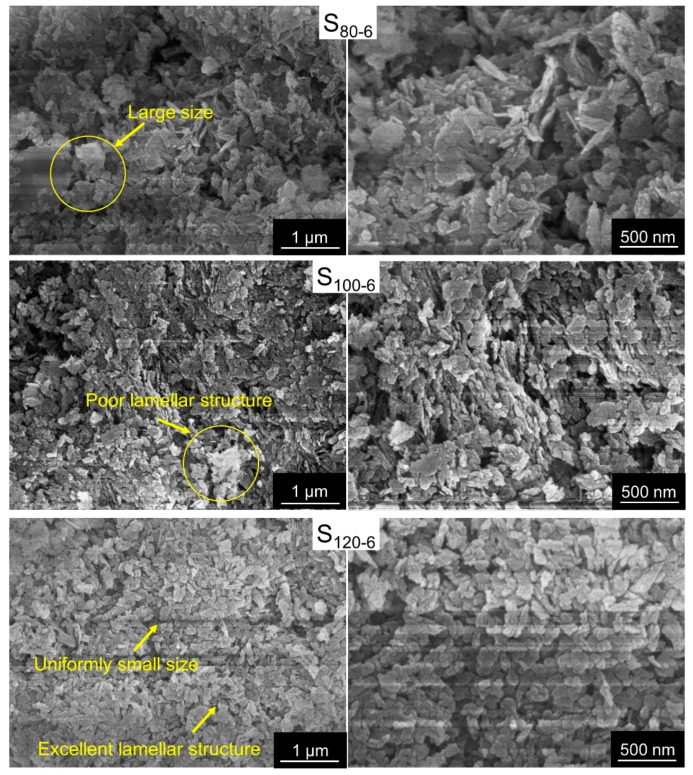
SEM images of CaAl-PO_4_-LDHs prepared using different crystallization temperatures.

The microstructures of the CaAl-PO_4_-LDHs were revealed via SEM. As shown in Figure 4, the samples synthesized at different crystallization temperatures had corresponding lamellar structures. The sample with a crystallization temperature of 80 °C had an excellent lamellar structure, but the sample had poor layer order and a large size. Via crystallization at 100 °C, the resulting samples exhibited small particle sizes but with poor lamellar structures. The samples synthesized at a crystallization temperature of 120 °C had a uniform shape, uniform particle size and excellent lamellar structure. Therefore, 120 °C is a better crystallization temperature for CaAl-PO_4_-LDH samples.

**Figure 5:**
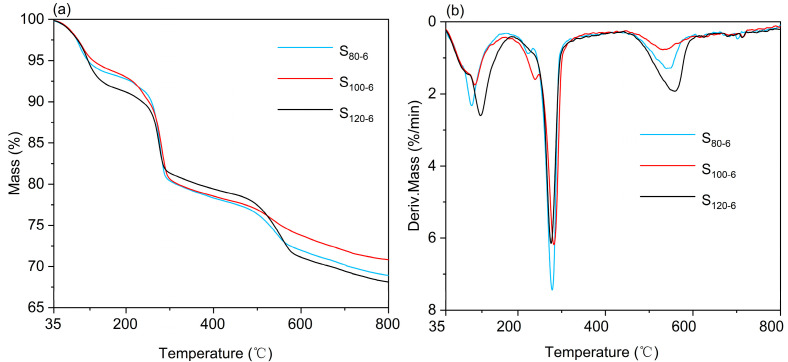
TG (**a**) and DTG (**b**) of CaAl-PO_4_-LDHs prepared using different crystallization temperatures in N_2_ atmosphere.

The CaAl-PO_4_-LDH samples were investigated via TG-DTG in N_2_ atmosphere, where the TG-DTG curves showed some differences. Figure 5 shows that the CaAl-PO_4_-LDH samples prepared at different crystallization temperatures all exhibited three thermal weight loss stages [30]. The first thermal weight loss stage (35–174 °C) is the loss of interlayer water molecules [31]. The S_120-6_ showed the highest thermal weight loss (9.18%) compared to S_80-6_ (6.66%) and S_100-6_ (6.23%), indicating that the sample had the highest number of water molecules in the interlayer. In addition, small amounts of anions may be released in the first stage [32]. The second stage (181–342 °C) of thermal weight loss is the detachment of -OH from the surface of CaAl-PO_4_-LDHs [33]. The weight loss of the S_120-6_ (11.75%) was lower than the other two samples (13.43% and 11.40%), which indicated that the layered structure was stable and numerous hydroxyl groups were not lost. The third thermal weight loss stage (450–629 °C) is a pyrolysis reaction via the anions in the interlayer [34]. In the third thermal weight loss phase, the S_120-6_ showed the highest thermal weight loss (8.63%), showing the sample contains the most interlayer anions. Furthermore, the thermal weight loss temperature of the S_120-6_ was slightly higher than that of the other two samples. Combined with the analysis of previous works, 120 °C is a better crystallization temperature for the preparation of CaAl-PO_4_-LDH samples via the coprecipitation method.

### 2.3. Effect of the Crystallisation Time on CaAl-PO_4_-LDHs

Based on the above analysis, the optimal crystallization temperature of 120 °C was determined, and then, the effects of different crystallization reaction times (4 h, 6 h and 8 h) on LDHs were investigated. The structures of CaAl-PO_4_-LDHs were investigated via FTIR, SEM, EDX and TG.

**Figure 6:**
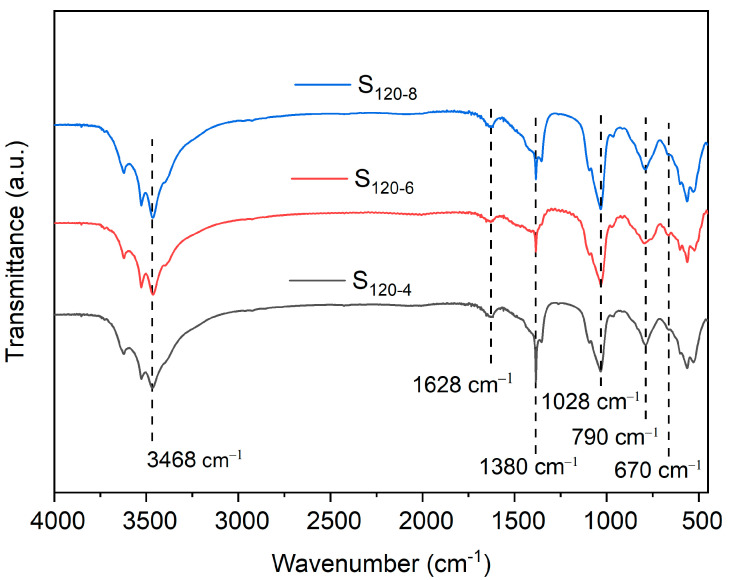
FTIR spectra of CaAl-PO_4_-LDHs prepared at different crystallization times.

The FTIR spectra of the CaAl-PO_4_-LDHs samples synthesized at different crystallization times had similar positions of signals (Figure 6). The signals near 3468 cm^−1^ were related to the hydroxyl stretching vibration in hydrotalcites. The signal at 1628 cm^−1^ was related to the bending vibration of the H-O-H [14]. The characteristic signals of CO_3_^2−^ corresponded to 1380 cm^−1^ and 790 cm^−1^ [24]. The S_120-6_ had almost no characteristic absorption band of CO_3_^2−^, indicating the sample was less oxidized. The signals at 1028 cm^−1^ were caused by the vibration of the PO_4_^3−^, showing PO_4_^3−^ was successfully embedded in the calcium–aluminum hydrotalcites [26]. The signals below 670 cm^−1^ were caused by O-M-O bonds and M-O (where M is Ca or Al) bonds [25].

**Figure 7:**
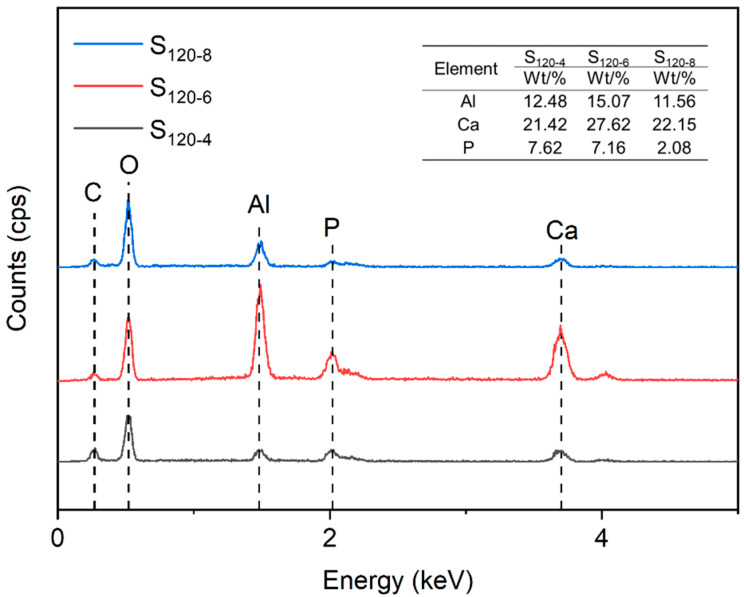
EDX spectra of CaAl-PO_4_-LDHs prepared at different crystallization times.

Figure 7 shows the EDX spectra of CaAl-PO_4_-LDHs samples prepared at different crystallization times. The values of the relative contents of Ca and Al elements of all CaAl-PO_4_-LDHs samples were close to the theoretical value of 2.0, indicating the successful synthesis of hydrotalcite structures [28]. With the increase in the crystallization time, the relative content of Ca and Al elements increased and then decreased, which revealed that the most LDHs were successfully synthesized when the crystallization time was 6 h. The high content of P elements in the samples synthesized at 6 h contributed to the higher content of PO_4_^3−^ being successfully inserted into the interlayer of LDHs [29].

**Figure 8:**
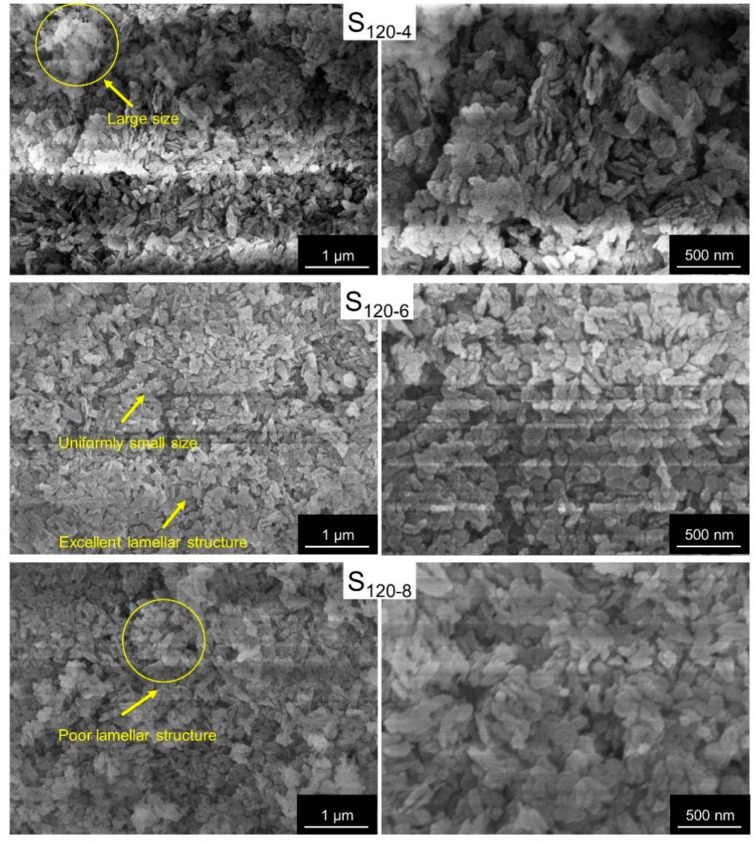
SEM of CaAl-PO_4_-LDHs prepared via different crystallization times.

The microstructures of the CaAl-PO_4_-LDH samples prepared at different crystallization times were determined via SEM, and the results are shown in Figure 8. All the CaAl-PO_4_-LDH samples indicated layered structures, which confirmed that the samples had typical structures of hydrotalcites. At the crystallization temperature of 120 °C, the morphological changes in the samples were not obvious with the increase in the crystallization time. However, the samples with crystallization times of 8 h exhibited some tendency toward lamellar structures to aggregate.

**Figure 9:**
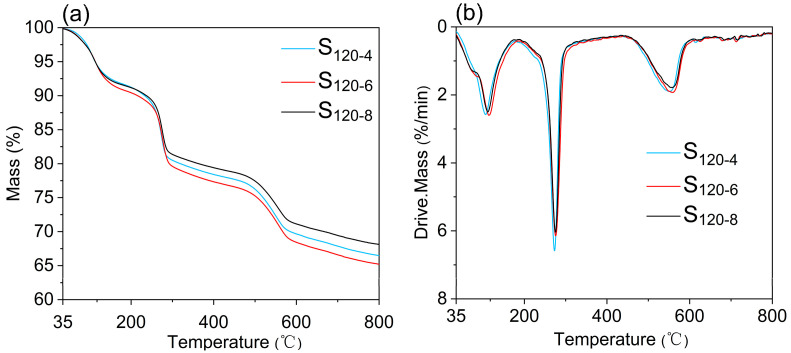
TG (**a**) and DTG (**b**) of CaAl-PO4-LDHs prepared via different crystallization times in N_2_ atmosphere.

Figure 9 shows that the thermal weight loss pattern of the synthesized samples was consistent with the three stage thermal weight loss pattern of hydrotalcites [30,35]. The sample synthesized at 6 h (S_120-6_) exhibited greater thermal weight loss (8.63%) than the others, S_120-4_ (8.20%) and S_120-8_ (7.78%), which indicates that the relative contents of -OH and PO_4_^3−^ in these samples were higher than those in the other samples. In addition, the initial pyrolysis temperature of the sample prepared via crystallization for 6 h was slightly higher than the other samples. All the results were compared and analyzed to conclude that the better crystallization time for the synthesis of CaAl-PO_4_-LDHs via coprecipitation was 6 h.

### 2.4. Effect of Different Concentrations of Flame-Retardant CaAl-PO_4_-LDHs on Bamboo Scrimber

From conclusions from the previous work, CaAl-PO_4_-LDHs were prepared at the optimum crystallization temperature (120 °C) and time (6 h) to be used as flame retardants for bamboo scrimber. The heat release rate (HRR), Time to Ignition (TTI) and Mass of Residue (Mass) of the bamboo scrimber treated with different concentrations of impregnated flame retardant were investigated.

**Figure 10:**
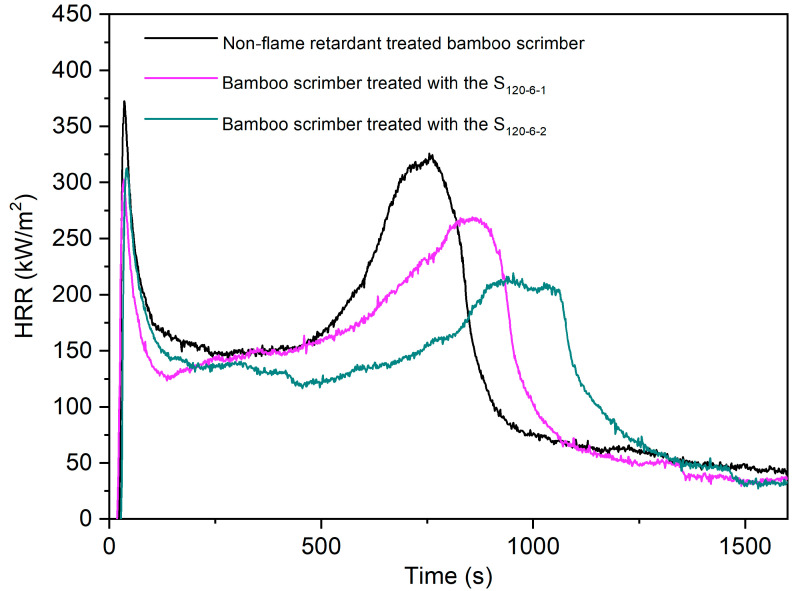
HRR of mass of non-flame-retardant-treated bamboo scrimber and flame-retardant bamboo scrimber.

The heat release rate (HRR) can reflect the speed and magnitude of heat released from a fire source during the combustion process of a material [36]. As can be seen from Figure 10, the intensity of exothermic peaks of the bamboo scrimber treated with the S_120-6-1_ and the bamboo scrimber treated with the S_120-6-2_ were smaller than the non-flame-retardant-treated bamboo scrimber. The first exothermic peak started at 30 s, and it corresponded to a short flaming combustion process when the sample was ignited. The second exothermic peak corresponded to the combustion process at the second appearance of the higher flame [37]. The second exothermic peak intensities of the bamboo scrimber treated with the S_120-6-1_ and the bamboo scrimber treated with the S_120-6-2_ were 17.58% and 34.46% lower than that of the non-flame-retardant-treated bamboo scrimber, respectively. Secondly, the arrival time of the exothermic peak was delayed by 103 s and 204 s for the bamboo scrimber treated with the S_120-6-1_ and the bamboo scrimber treated with the S_120-6-2_, respectively. The results indicate that the strong fire arrival time during combustion was delayed for the bamboo scrimber treated with the flame-retardant CaAl-PO_4_-LDHs, so the flame retardancy of bamboo scrimber was enhanced. The Time to Ignition (TTI) is the time required to produce continuous combustion on the surface of a sample due to thermal radiation [38]. A smaller TTI suggests higher combustibility. The TTIs of the control group and bamboo scrimber treated with different concentrations of impregnated flame-retardant CaAl-PO_4_-LDHs are shown in Table 2. Compared to the non-flame-retardant-treated bamboo scrimber, the TTIs of the bamboo scrimber treated with the S_120-6-1_ and the bamboo scrimber treated with the S_120-6-2_ were delayed by 30% and 40%, respectively, suggesting the heat resistance of the bamboo scrimber was improved. Furthermore, the bamboo scrimber treated with the S_120-6-2_ exhibited a higher deferral rate of TTI, showing better flame retardancy.

**Figure 11:**
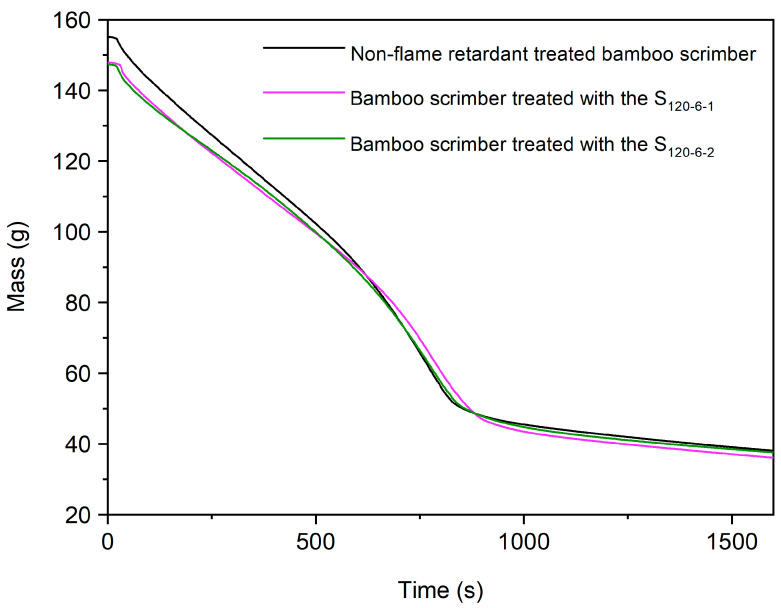
Mass of non-flame-retardant bamboo scrimber and flame-retardant bamboo scrimber.

The Mass of Residue (Mass) allows for the visual analysis of the stability of a material at high temperatures. The Mass of Residues of the control group and bamboo scrimber treated with different concentrations of flame retardants are shown in Figure 11. The residual carbon rate indicated the final residual mass of the bamboo scrimber as a percentage of the initial mass. The residual carbon rates of the non-flame-retardant-treated bamboo scrimber, the bamboo scrimber treated with the S_120-6-1_ and the bamboo scrimber treated with the S_120-6-2_ were 22.24%, 23.12% and 24.32%, respectively. The results showed that CaAl-PO_4_-LDHs did not have a significant effect on the residual carbon rate of the bamboo scrimber and only slightly increased its residual carbon rate.

Materials produce many toxic gases during combustion, and the control of these gases is essential. As shown in Table 3, compared with non-flame-retardant-treated bamboo scrimber, the average CO production of bamboo scrimber treated with the S_120-6-1_ and bamboo scrimber treated with the S_120-6-2_ decreased by 18.87% and 26.42%, respectively, and the average arrival time of the maximum CO peak was delayed by 297 s and 253 s, respectively. In addition, the average CO_2_ production decreased by 11.11% and 14.46%, respectively, and the average arrival time of the maximum CO_2_ peak was delayed by 213 s and 268 s, respectively. These results indicate that CaAl-PO_4_-LDHs can suppress the amount of CO and CO_2_ production during bamboo combustion, and the delayed maximum peak arrival time can buy rescue time when a fire occurs.

**Figure 12:**
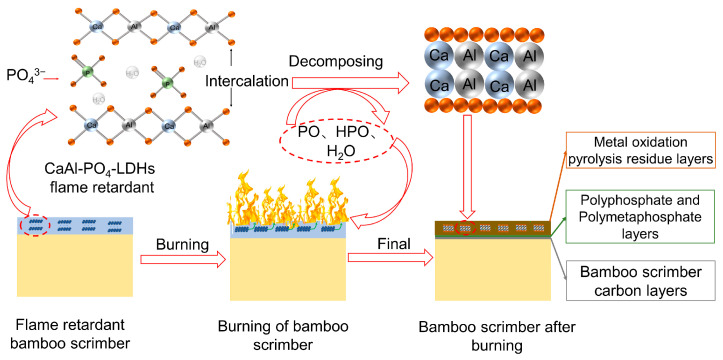
Possible flammability and charring process of CaAl-PO_4_-LDH-treated bamboo scrimber.

As shown in Figure 12, LDHs make bamboo produce char residue more easily, which can isolate O_2_ and heat transfer between burning areas and the bottom of the carbon layer. During the combustion of CaAl-PO_4_-LDHs, hydroxyl groups on layers and interlaminar anions are released in the form of H_2_O and CO_2_, which can adsorb a lot of heat and reduce the concentration of combustion gas [39,40]. Thus, the heat release rate can be slowed down, and the ignition time of reconstituted bamboo can be delayed. Additionally, the final pyrolysis residue of CaAl-PO_4_-LDHs can also catalyze the formation of a more stable carbon layer and cover the surface of bamboo. The physical process of the char residue acts as a protective barrier, resulting in improved flame retardancy for bamboo [41]. So, the flame resistance of recombinant bamboo scrimber can be improved in this way.

## 3. Materials and Methods

### 3.1. Materials

Calcium nitrate tetrahydrate (Ca(NO_3_)_2_·4H_2_O), aluminum nitrate tetrahydrate (Al(NO_3_)_3_·9H_2_O), sodium hydroxide (NaOH) and sodium phosphate (Na_3_PO_4_) were purchased from Sinopharm Chemical Reagent Co., Ltd., Shanghai, China. All chemicals were of analytical grade. All solutions in the experiment were prepared with deionized water.

### 3.2. Preparation of CaAl-PO_4_-LDHs via Coprecipitation

In this study, CaAl-PO_4_-LDHs were prepared via the coprecipitation method at 80 °C, 100 °C and 120 °C, and the crystallization times were 4 h, 6 h and 8 h. The pH value was kept at 10.0–11.00. In detail, we mixed Ca(NO_3_)_2_·4H_2_O and Al(NO_3_)_3_·9H_2_O with a $\frac{M^{2+}}{M^{3+}}$ molar cationic ratio of 2.0/1.0 (solution A) and prepared the solution of a strong base which was 1.5 mol/L NaOH. Then, solution A and NaOH solution were added into constant pressure funnels. A total of 41 g of Na_3_PO_4_ was dispersed in 500 mL of deionized water in a 1000 mL three-necked flask before being stirred with a magnetic stirrer. After that, the reaction solution was put in 25 °C conditions for 16 h. Finally, the reaction solution was prepared via extraction, washing and drying to obtain CaAl-PO_4_-LDHs. Each group of experiments was repeated three times.

### 3.3. CaAl-PO_4_-LDH Flame-Retardant-Treated Bamboo Scrimber

The CaAl-PO_4_-LDH flame retardant was ultrasonically dispersed in an aqueous solution at 25 °C and prepared to suspensions of 1% and 2%. Then, the bamboo scrimber was dipped in the CaAl-PO_4_-LDH suspension and impregnated for 2 h at atmospheric pressure with simultaneous stirring [12]. After the impregnation, the suspended matter was removed from the surface of the bamboo scrimber samples with deionized water, and the bamboo scrimber samples were dried in an oven.

**Figure 13:**
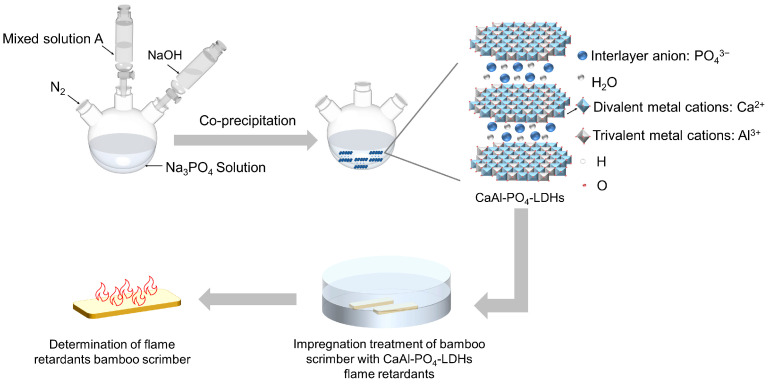
The process of synthesizing CaAl-PO_4_-LDHs and preparing flame-retardant bamboo scrimber.

A summary of the CaAl-PO_4_-LDH experimental samples is shown in Table 4. Figure 13 illustrates the process of synthesizing CaAl-PO_4_-LDHs and preparing flame-retardant bamboo scrimber.

### 3.4. Characterization

X-ray diffraction (XRD) was carried out on an XRD-D2 produced by the German Brooke Company (Bremen, Germany). The scanning range was 5–70° (2*θ*) and the scanning speed was 6°/min. The samples were analyzed via FTIR spectroscopy using a Prestige-21 instument (Shimadzu Corporation, Shimane, Japan) with a scanning range of 400–4000 cm^−1^. The potassium–bromide pellet method was used to determine the chemical composition of the samples prepared under different conditions (the samples were dried in an oven at 80 °C, and the dried samples were mixed with potassium bromide in a 1:100 ratio for grinding). The morphology and dispersion of samples prepared under different conditions were observed via the SU8010-type cold field emission SEM produced by Hitachi, Japan. The sample elements were investigated in combination with SEM to detect the relative content of Ca, Al and P elements in the sample. The thermogravimetric analysis (TGA) was measured using TA Q6000 (Naichi Instrument Manufacturing GmbH, Selb, Germany) at the heating rate of 20 °C/min under N_2_ conditions with a temperature range of 35–800 °C and the flow rate of 40 mL/min. We determined the amount of charcoal residue in a sample by heating the sample to measure the weight change of the sample. Finally, the flame retardancy of the samples was tested using a conical calorimeter (CONE) manufactured by Nechi Instruments GmbH, Selb, Germany. According to the standard ISO 5660, the heat radiation power was 50 kW/m^2^ and the sample size was 100 mm × 100 mm × 3 mm^3^. The results were obtained from the average of three replicates. All samples were wrapped in aluminum foil with no cover on the upper surface. The samples were then placed in a holder and exposed horizontally to reduce heat spillage to the outside during combustion.

## 4. Conclusions

In this work, PO_4_^3−^-anion-intercalated calcium–aluminum hydrotalcites were successfully synthesized via the coprecipitation method. The microstructure and thermal stability were confirmed using XRD, FT-IR, SEM, EDX and TG, and the optimal crystallization temperature and time for the synthesis of CaAl-PO_4_-LDHs were investigated. The results indicated that the optimal crystallization temperature for the synthesis of CaAl-PO_4_-LDHs via coprecipitation was 120 °C and the optimal crystallization time was 6 h, and the PO_4_^3−^ anion was also successfully intercalated into the interlayer of calcium–aluminum hydrotalcites.

Different concentrations (1% and 2%) of CaAl-PO_4_-LDHs were used as flame retardants for the bamboo scrimber, and the flame retardancy of the bamboo scrimber was evaluated via HRR, TTI and Mass. The results show that the presence of CaAl-PO_4_-LDHs improved the fire resistance of the bamboo scrimber. Compared with bamboo scrimber without CaAl-PO_4_-LDHs, the HRR peaks of bamboo scrimber treated with 1% and 2% concentrations of CaAl-PO_4_-LDHs were reduced by 16.62% and 34.46%, respectively, and the time taken to reach the exothermic peak was delayed by 103 s and 204 s, respectively. The TTI increased by 6 s and 8 s, respectively. The residual carbon rate of the bamboo scrimber did not change significantly. The average CO production decreased by 18.87% and 26.42%, respectively, and the average CO_2_ production decreased by 11.11% and 14.46%, respectively. This work shows the possibility of PO_4_^3−^ ions being successfully intercalated into the interlayer of calcium-aluminum hydroxides and the potential of CaAl-PO_4_-LDHs for flame retardant applications in bamboo scrimber.

## Figures and Tables

**Table 1:** Layer spacing parameters for CaAl-PO_4_-LDHs samples and PDF#50-0652.

Sample	d_003_ (Å)	d_006_ (Å)	d_110_ (Å)
S_80-4_	8.54	4.30	2.78
S_80-6_	8.56	4.30	2.77
S_80-8_	8.63	4.32	2.87
S_100-4_	8.61	4.32	2.87
S_100-6_	8.80	4.42	2.87
S_100-8_	8.73	4.35	2.87
S_120-4_	8.71	4.34	2.89
S_120-6_	8.68	4.30	2.87
S_120-8_	8.64	4.31	2.85
PDF#50-0652	8.60	4.31	2.87

**Table 2:** Mean parameter values of the samples.

Sample	Mean			
	HRR (kW/m^2^)	pHRR (kW/m^2^)	TTI (s)	Mass Reduction (%)
Non-flame-retardant-treated bamboo scrimber	122.95 ± 3.65	359.99 ± 12.40	20 ± 0	22.63 ± 0.01
Bamboo scrimber treated with the S_120-6-1_	117.41 ± 1.44	310.71 ± 8.23	26 ± 6	21.12 ± 0.73
Bamboo scrimber treated with the S_120-6-2_	115.74 ± 0.62	324.21 ± 11.61	28 ± 1	24.32 ± 0.71

**Table 3:** The samples’ released parameters of CO and CO_2_.

Sample	CO Yield			CO_2_ Yield		
	Mean (kg/kg)	Peak (kg/kg)	Time to Peak (s)	Mean (kg/kg)	Peak (kg/kg)	Time to Peak (s)
Non-flame-retardant-treated bamboo scrimber	3.10 ± 0.08	49.54 ± 1.36	1116 ± 116	4.46 ± 0.10	90.06 ± 10.47	854 ± 14
Bamboo scrimber treated with the S_120-6-1_	2.51 ± 0.03	46.5 ± 3.34	1413 ± 156	3.96 ± 0.01	90.18 ± 8.66	1067 ± 6
Bamboo scrimber treated with the S_120-6-2_	2.281 ± 0.13	46.81 ± 0.13	1369 ± 134	3.81 ± 0.25	93.92 ± 5.19	1122 ± 45

**Table 4:** CaAl-PO4-LDH experimental samples.

Sample Number	Crystallization Temperatures	Crystallization Times	Mass Percentage Concentration
S_80-4_	80 °C	4 h	-
S_80-6_	80 °C	6 h	-
S_80-6_	80 °C	8 h	-
S_100-4_	100 °C	4 h	-
S_100-6_	100 °C	6 h	-
S_100-8_	100 °C	8 h	-
S_120-4_	120 °C	4 h	-
S_120-6_	120 °C	6 h	-
S_120-8_	120 °C	8 h	-
S_120-6-1_	120 °C	6 h	1%
S_120-6-2_	120 °C	6 h	2%

## Data Availability

Not applicable.

## Sample Availability

Not available.
